# Human Gait Analysis Metric for Gait Retraining

**DOI:** 10.1155/2019/1286864

**Published:** 2019-11-11

**Authors:** Tyagi Ramakrishnan, Seok Hun Kim, Kyle B. Reed

**Affiliations:** University of South Florida, USA

## Abstract

The combined gait asymmetry metric (CGAM) provides a method to synthesize human gait motion. The metric is weighted to balance each parameter's effect by normalizing the data so all parameters are more equally weighted. It is designed to combine spatial, temporal, kinematic, and kinetic gait parameter asymmetries. It can also combine subsets of the different gait parameters to provide a more thorough analysis. The single number quantifying gait could assist robotic rehabilitation methods to optimize the resulting gait patterns. CGAM will help define quantitative thresholds for achievable balanced overall gait asymmetry. The study presented here compares the combined gait parameters with clinical measures such as timed up and go (TUG), six-minute walk test (6MWT), and gait velocity. The comparisons are made on gait data collected on individuals with stroke before and after twelve sessions of rehabilitation. Step length, step time, and swing time showed a strong correlation to CGAM, but the double limb support asymmetry has nearly no correlation with CGAM and ground reaction force asymmetry has a weak correlation. The CGAM scores were moderately correlated with TUG and strongly correlated to 6MWT and gait velocity.

## 1. Introduction

Researchers traditionally analyze a small set of gait parameters in order to evaluate the outcomes of their techniques. This often leads to an overreliance on a few parameters and a focus on improving one gait parameter. Few studies in the gait literature aim to correct many gait parameters at the same time. This traditional narrow approach lacks broader understanding of the interaction between various gait parameters and limits potential approaches that can lead to wholesome rehabilitation techniques. In this research study, we examine our combined gait asymmetry metric (CGAM) to give a representation of the overall gait pattern. We use stroke for examining this combined metric because it affects several different aspects of an individual's gait, and many of these aspects are asymmetric. Although we focus on measures of asymmetry, this combined method is not limited by the type or number of parameters evaluated. Our hypothesis is that the outcomes of the combined metric will partially correlate to functional clinical outcome measures. We also use this combined metric to determine if there have been changes to the individual's gait pattern from baseline to after the clinical intervention.

**Figure 1:**
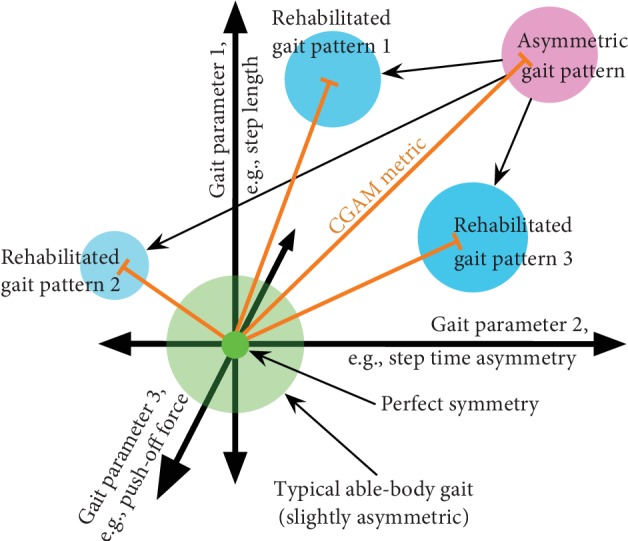
Representation of the multidimensional gait parameter space. The orange lines represent the distance each gait is from a symmetric gait (CGAM distance), which helps determine how far away a gait is from ideal. CGAM can also aid in ascertaining whether the overall gait pattern is improving (even if some of the parameters are getting worse). CGAM can incorporate more dimensions than the three shown, but that is hard to visualize.

Figure 1 shows an example of how a combined metric would be useful in analyzing an asymmetric gait pattern. Many existing rehabilitation therapies can change different sets of gait parameters, but some make one parameter worse while correcting others. Even in unimpaired walking, perfect symmetry is not expected [1], so there is space for some parameters to be asymmetric while the overall gait is within a reasonable bound. The CGAM distance (shown in orange in Figure 1) generates a single representation of the measured gait parameters that generally scales with the global deviation from symmetry. The deviation of each measure is scaled based on the variance within that measure, so measures that generally have larger magnitudes of asymmetry (e.g., forces) will be scaled so that each gait parameter has a similar influence on the overall metric. If a therapy reduces the CGAM distance, the overall gait has improved even though some of the individual parameters might have gotten worse. Without a combined metric, it is difficult to determine whether the gait is improving when looking at individual gait parameters.

### 1.1. Gait Measurements

Gait data is typically collected using motion capture, force plates, and/or wearable sensors. Many variables portray various facets of human gait. There are spatial parameters such as step length defined by the distance covered from the heel strike of one foot to the heel strike of the opposite foot. There are temporal parameters such as step time defined as the time taken between opposite heel strikes. Then, there is swing time, which is the time taken from toe-off to heel strike of the same foot. Double limb support is the time spent when both legs are on the ground. The terminal double limb support is used for this research study. There are kinematic parameters associated with joint angles of the ankle, knee, and hip joints. Hip joints in the case of individuals with stroke and amputees also show abduction and adduction. The kinetic parameters include vertical ground reaction forces, propulsive or push-off forces during toe-off, braking forces during initial contact or heel strike, and ankle, knee, and hip joint moments. Further, some of these parameters are more easily identified by sight alone (e.g., step length, cadence, and gait velocity) while others are nearly impossible to quantify without a sensor (e.g., forces and joint moments) [2].

### 1.2. Gait Metrics

Several gait metrics combining multiple gait parameters have been used clinically to evaluate different gait impairments. These metrics can also be used to classify gait based on different types of information. There are two types: qualitative [3, 4] and quantitative [5–7] metrics. Many metrics rely on either kinetic or kinematic data to categorize different gait motions and behaviors. Some metrics have the ability to jointly analyze kinetic and kinematic parameters [8, 9]. Machine learning has been used to classify and differentiate gait patterns [10]. Most gait metrics use statistical analysis like principle component analysis (PCA) and singular variable decomposition (SVD) to reduce dimensionality to make the data computation easier [11]. The processed data is then classified using the Euclidean or similar distances [11]. These distances become the scores which form the central part of the gait metric. Another study by Hoerzer et al. [9] proposed the comprehensive asymmetry index (CAI) which combined gait asymmetry using PCA and Euclidean distances. CAI was effective in identifying that running with shoes reduces gait asymmetry compared to barefoot running. A prior study used a combination of Mahalanobis distances with data reduction techniques on a preprocessed dataset to analyze kinematic and kinetic gait parameters [8]. They developed several metrics to classify the data and showed that they can successfully classify the abnormal data from a standard normal dataset. The precursor to CGAM used a symmetry index processed using PCA measured using Mahalanobis distances. Without the restrictions of dimensionality reduction, CGAM served as a versatile gait asymmetry metric [12–14].

### 1.3. Effects of Stroke on Gait and Rehabilitation

The analysis in this paper uses an existing dataset from an experimental stroke therapy to examine the effects of combining and jointly assessing gait as opposed to individually assessing a single parameter. We focus on individuals with stroke because they inherently have different capabilities on each side and are asymmetric; as such, it is unlikely that they can ever regain complete symmetry in all parameters. However, it may be possible to achieve a balanced gait where some parameters are slightly asymmetric, but none of them are excessively large. Our proposed joint metric helps to balance all of the parameters. We examine before and after the therapy to help understand what changes have occurred.

Gait after stroke becomes asymmetric (or hemiparetic) as a consequence of altered neuromuscular signals affecting leg motor areas, typically hyperextension at the knee and reduced flexion at the hip, knee, and ankle [15–17]. Hemiparetic gait is characterized by significant asymmetry in temporal (e.g., time spent in double limb support) and spatial (e.g., step length) measures of interlimb coordination [15, 18, 19]. Propulsive force of the paretic limb is reduced compared to the nonparetic limb, as are work and power of the paretic plantar flexors [19, 20]. The significant decrease in propulsive force results in smaller overall step lengths, which in turn affects the patient's gait velocity. Finally, vertical ground reaction forces (GRFs) are decreased on the paretic limb relative to the nonparetic limb [21], reflecting diminished weight bearing and balancing capabilities by the paretic limb.

Some of the rehabilitation techniques used to restore gait impaired by stroke involve some form of asymmetric perturbations that try to restore the symmetry between the paretic and nonparetic sides [22]. Split-belt treadmills are one method to apply this rehabilitation technique. The split-belt treadmill has two treads that can move at different velocities, which are used to exaggerate the asymmetry of the individual. When the tread speeds are made the same after training, the subject typically has some after-effects that are more symmetric than when they started [23]. The after-effects are usually improved spatial and temporal symmetry. Unfortunately, these after-effects only partially transfer to walking on the ground. There are other rehabilitation techniques such as body-weight support [24], robotic [25], functional electrical stimulation [26], transcranial magnetic stimulation [27], and full-body gait exoskeletons [28]. Each of the techniques have their merits and train the individual in a specialized manner, which means a combination of these methods may provide additional benefits to the person.

## 2. CGAM Derivation

The metric presented here has the potential to help categorize and differentiate between multiple asymmetric gaits [29]. CGAM is based on Mahalanobis distances, and it utilizes the asymmetries of gait parameters obtained from data recorded during human walking. The gait parameters that were used in this analysis represent spatial, temporal, and kinetic parameters. This form of a consolidated metric will help researchers identify overall gait asymmetry and improve rehabilitation techniques to provide a well-rounded gait post training. The CGAM metric successfully served as a measure for overall symmetry with 11 different gait parameters and successfully showed differences among gait with multiple physical asymmetries [14]. The mass at the distal end had a larger magnitude on overall gait asymmetry compared to leg length discrepancy. Combined effects are varied based on the cancellation effect between gait parameters [13]. The metric was successful in delineating the differences of prosthetic gait and able-bodied gait at three different walking velocities [14].

Symmetry is calculated using equation (1) where *M* is the step length, step time, swing time, double limb support (DLS), and ground reaction forces (GRFs). A value of 0 indicates symmetry. The measures include gait evaluations conducted before training and after the completion of training.

(1) $$\begin{array}{c} \text{Symmetry}=100*\frac{\text{abs}\left(M_{\text{paretic}}-M_{\text{nonparetic}}\right)}{0.5*\left(M_{\text{paretic}}+M_{\text{nonparetic}}\right)}, \end{array}$$

(2) $$\begin{array}{c} \text{Modified CGAM}=\sqrt{\frac{\text{Data}*\text{inv}\left(\Sigma \right)*{\text{Data}}^{\prime }}{\sum \left(\text{inv}\left(\Sigma \right)\right)}}, \end{array}$$

where

- Modified CGAM distance: weighted distance from ideal symmetry
- CGAM distance: Mahalanobis distance from ideal symmetry
- Data: matrix with *n* columns (11) and *m* rows (number of steps)
- *Σ*: covariance of the data

The modified CGAM [30] works similar to weighted means, but, in this case, the weights are inverse covariances that are multiplied across the dataset in the numerator. To balance the influence of the inverse of covariance, it is divided by the sum of the inverse covariance matrix, equation (2). This change to the formulation makes the modified CGAM represent the scores closer to the percent asymmetry while still serving as a combined measure of all the gait parameter asymmetries.

## 3. Methods

The analysis performed in this paper used data collected as part of a separate clinical study. The novel shoe tested was designed to improve the overall gait symmetry and gait function of an individual poststroke. The efficacy of the device is discussed in another paper [31]. That study data is used here so we can evaluate the modified CGAM in the context of a rehabilitation therapy. This study aims to understand how the modified CGAM metric can be used to evaluate the gait of individuals with stroke. The study data consists of six subjects who trained on the device for four weeks. Gait parameters and functional clinical measures were collected throughout the training and used in the modified CGAM analysis presented here.

### 3.1. Subjects

All subjects agreed to participate in this study and signed a consent form that was approved by the Western Institutional Review Board. Six subjects (4 males and 2 females), aged 57–74 years old with unilateral stroke, completed the training, and the length of time since stroke ranged from 1.2 to 12.5 years. Subject 3 was an outlier and excluded in some of the analyses. At baseline, his double limb support asymmetry was 34 standard deviations above the other subjects' mean and timed up and go (TUG) score was 36 standard deviations above the other subjects' mean.

### 3.2. Device Used for Gait Training

**Figure 2:**
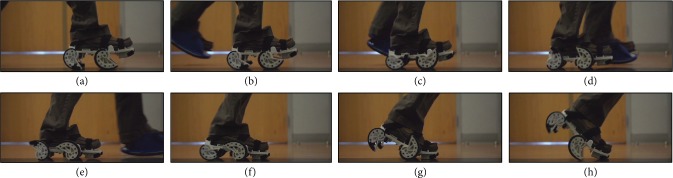
As the wearer takes a step, the device pushes the foot backward during stance. This exaggeration of the asymmetry results in a more symmetric gait pattern once the shoe is removed. In addition, the shoe works to strengthen the paretic leg by slightly destabilizing the nonparetic leg, which encourages the wearer to use their paretic leg more. A flexible height- and weight-matched platform worn on the opposite foot equalizes the added height and weight of the device.

The device, shown in Figure 2, is designed to change interlimb coordination and strengthen the paretic leg of individuals with asymmetric walking patterns caused by stroke. The concept of this device is similar to that of a split-belt treadmill [32] but allows the individual to walk over ground, which is hypothesized to help with long-term retention of the altered gait pattern [33]. The device is completely passive and uses spiral-like (nonconstant radius) wheels [34], which redirect the downward force generated during walking into a backward force that generates a consistent motion. By not utilizing actuators and fabricating the shoe using rapid manufactured glass-filled nylon, the version used in this study weighs approximately 900 g. Small unidirectional dampers on the front and back axles prevent uncontrolled motions. After the shoe stops moving backward, the user pushes off, and springs attached to the axles reset the position of the wheels for the next step. The front of the device is able to pivot to more naturally conform to the user's toe-off.

### 3.3. Experiment Procedure

Before training, the subject's gait patterns were evaluated using a ProtoKinetics Zeno Walkway (ProtoKinetics, Havertown, PA). They then completed four weeks of training three times a week under the guidance of a physical therapist. Each of the twelve sessions included six bouts of walking for five minutes on the device with about a two-minute break between bouts. The device was attached to the subject's nonparetic foot during training. The subject's gait without the device was measured on the ProtoKinetics Zeno Walkway before the training began [35]; this data will be referred to henceforth as pretest. Gait data was also collected on the walkway prior to the second, third, and fourth week of training sessions; this data will be referred to as midtest. Their gait was tested again within five days after the completion of the training protocol on the walkway; this data will be referred to as post test. Clinical measures included TUG [36], six-minute walk test (6MWT) [37], and gait velocity.

### 3.4. Data Analysis

The modified CGAM scores for all the trials were calculated using spatial, temporal, and kinetic parameter asymmetries. The *R*-squared (*r*^2^) was used to assess the correlations between the modified CGAM scores and clinical measures. The correlations between the clinical measures and individual gait parameters were also analyzed using *r*^2^. The strength of correlation was evaluated based on the absolute value of *r* as reported by Swinscow et al. [38] where *r* = 0.4 and above is moderate or strong correlation.

## 4. Results

**Figure 3:**
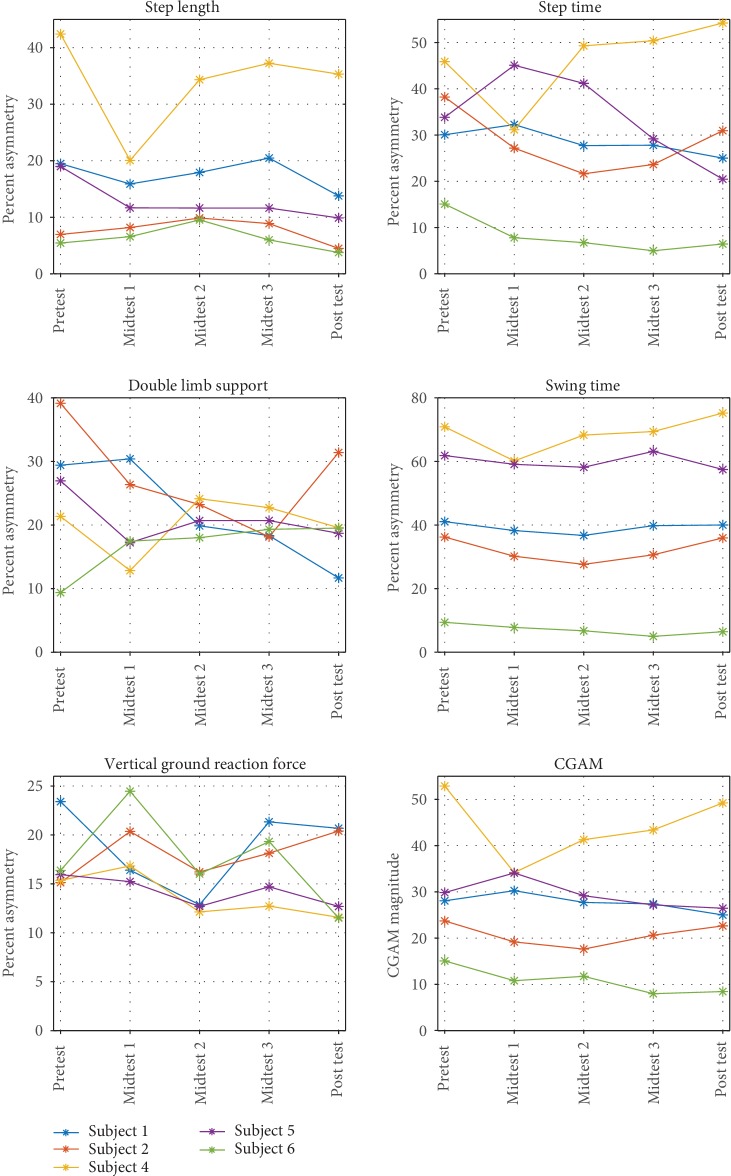
Gait parameter asymmetry.

The individual gait parameter asymmetries are shown in Figure 3 for reference. Details related to the results from the clinical trial are presented in another paper [31]. The below results focus on the modified CGAM.

Table 1 shows the correlation values between the pre- and post test data of each gait parameter for all subjects correlated with the corresponding modified CGAM scores. The pre- and post test performance is important clinically; however it is also important to analyze the correlation for all the midtest data points for the gait parameters, so both time frames are shown. It is interesting to note that step length, step time, and swing time show consistently very strong correlation to the modified CGAM while double limb support asymmetry shows a very weak correlation. The correlations between step length, step time, swing time, and double limb support remain consistent between the pre-/post comparison and data from all weeks. The ground reaction force has a stronger correlation for all midtests compared to just the pre- and post tests.

Table 2 shows the complete list of *r*^2^ values comparing the gait parameters and modified CGAM to the functional gait measures. Modified CGAM scores show a moderate correlation to TUG and strong correlations with 6MWT and gait velocity. Step time and swing time asymmetries show a similar pattern of correlation as the modified CGAM does. TUG shows a moderate correlation to step time, swing time, and ground reaction force asymmetries, but weak and very weak correlations to step length and double limb support asymmetries, respectively. The 6MWT and gait velocity show moderate correlations to step length asymmetry and strong correlations to step time and swing time asymmetries, but weak correlations to double limb support and ground reaction force asymmetries.

## 5. Discussion

Comparing the behavior of the gait parameters helps understand the relationship between the gait asymmetries and also evaluates the hypothesis that there exists a balance of asymmetry between gait parameters. For example, most subjects in midtest 1 show a decrease in spatial and temporal asymmetry but have increases in ground reaction force asymmetry. The reverse is observed in midtest 2 where most subjects have decreased ground reaction force but increased spatial and temporal asymmetry. Not all subjects display the same changes, but this highlights the difficultly of determining if the overall gait improved or not since improving one gait parameter may come partially at the expense of making another gait parameter worse. People with hemiparesis due to stroke have different force and motion capabilities on each leg. The paretic leg is weaker and has a more limited range of motion than the nonparetic leg. Rehabilitation science has not advanced to the point where these problems can be fully corrected. Therefore, when we are retraining walking poststroke, we are working with an inherently asymmetric system. From a biomechanical view, two physically different systems (e.g., legs) can only have the same motion if the forces controlling them or the forces resulting from the movement are different. When an individual with an asymmetric impairment walks with symmetric step lengths, other aspects of gait become asymmetric, such as the forces in the joints [39, 40], the amount of time standing on each leg [21], and other temporal variables [41, 42], all of which can be detrimental to efficiency and long-term viability.

All subjects decreased the modified CGAM score, which indicates that their overall gait improved. This does not mean that every gait parameter improved. For example, subject 2 had slightly worse swing time and vertical ground reaction force asymmetries and subject 4 had slightly worse step time and swing time asymmetries during the post test compared to the pretest. But, the other gait parameters improved such that the end result was an overall better gait pattern. This suggests that there can be a functional balance between all the gait parameters. Although the resulting gait will have some degree of asymmetry in all measures, it will more likely meet the functional walking goals of individuals with asymmetric impairments.

The modified CGAM can be calculated using any number of input gait parameters. Including more should give a better indication of the overall gait, but care should be given to including a range of different types of parameters like forces, spatial, and temporal parameters. Also of note is that the specific score of modified CGAM with one set of parameters is not directly comparable to modified CGAM computed with a different set of parameters. So, modified CGAM can be very helpful for looking at changes within a study but may not always provide a comparison between studies if the measured parameters are different.

Modified CGAM shows a strong correlation with step length, step time, and swing time. This was consistent when only the pre- and post test data were considered or when all test data including pre- and post tests were analyzed. This means that these three parameters have similar behaviors to their modified CGAM scores while double limb support and ground reaction force asymmetry have more variation in the data.

The modified CGAM scores calculated using the spatial, temporal, and kinetic parameters showed behaviors similar to some of the underlying gait parameter asymmetries (see Figure 3) and also some of the functional measures. Although it would be expected to have some correlation to the underlying parameters, having moderate to strong correlation with the functional measures shows evidence that a measure of overall symmetry which is used as factor for gait quality is related to gait function signified by gait velocity and 6MWT. These findings also offer some evidence to validate the modified CGAM metric.

## 6. Conclusions

To summarize, the research suggests that rehabilitating gait asymmetries should be a holistic approach. Targeting certain types of asymmetry may not be the correct approach as it may adversely affect other gait parameters that may lead to pervasive long-term effects. The modified CGAM metric showed potential for being used as a quantitative metric for impairments that cause gait asymmetries. Further, the research suggests that it is important to consider quantitative metrics such as modified CGAM and subjective metrics such as pain and quality of life data to evaluate overall improvement of an individual's gait. The simple asymmetric perturbations applied on the gait patterns showed that it is possible to combat the negative effects of asymmetric impairment with asymmetry. To tackle these problems, this research has shown that quantitative metrics along with clinical evaluation offer a good direction in evaluating and rehabilitating asymmetric gait patterns.

## Figures and Tables

**Table 1:** Correlation (*r*^2^) between modified CGAM and gait parameters.

Gait parameter (asymmetry)	Modified CGAM (pre & post)	Modified CGAM (all midtests)
Step length	**0.93**	**0.81**
Step time	**0.95**	**0.88**
Swing time	**0.98**	**0.89**
Double limb support	0.01	0.01
Ground reaction force	0.03	**0.18**

Bold implies correlation that is moderate or above.

**Table 2:** Correlation (*r*^2^) between clinical measures and gait parameters.

Gait parameter	TUG	6MWT	Gait velocity
Step length asymmetry	0.14	**0.21**	**0.31**
Step time asymmetry	**0.23**	**0.53**	**0.63**
Swing time asymmetry	**0.29**	**0.43**	**0.57**
Double limb support asymmetry	0.03	0.14	0.10
Ground reaction force asymmetry	**0.26**	0.14	0.13
Modified CGAM	**0.22**	**0.41**	**0.51**

Bold implies correlation that is moderate or above.

## Data Availability

The data used to support the findings of this study are available from the corresponding author upon request.
